# 89 Pooled Safety Analysis Evaluating Bioengineered Allogeneic Cellularized Construct in Patients with Deep Partial-thickness Thermal Burns

**DOI:** 10.1093/jbcr/irac012.092

**Published:** 2022-03-23

**Authors:** James H Holmes, Leopoldo C Cancio, Lee D Faucher, Kevin N Foster, Angela Gibson

**Affiliations:** Atrium Health Wake Forest Baptist, Winston-Salem, North Carolina; US Army Institute of Surgical Research, JBSA Fort Sam Houston, Texas; University of Wisconsin, Madison, Wisconsin; The Arizona Burn Center Valleywise Health, Phoenix, Arizona; University of Wisconsin School of Medicine and Public Health, Madison, Wisconsin

## Abstract

**Introduction:**

Autograft (AG) is the standard of care treatment for deep burns but requires creation of a donor site wound prone to pain and scarring. Treatment with a bioengineered allogeneic cellularized construct (BACC) is an alternative approach that can reduce or eliminate the need for autografting. The BACC is a bilayer construct that was recently approved in the US for the treatment of adult deep partial-thickness (DPT) burns. Here, we report the analysis of pooled safety data from two open-label, randomized, controlled trials (STRATA2011 [NCT01437852] and STRATA2016 [NCT03005106]) that evaluated efficacy and safety of BACC versus autografting in patients with DPT burns.

**Methods:**

The trials enrolled 101 patients aged ≥18 years with 3–49% total body surface area (TBSA) thermal burns. In each patient, two DPT areas on the torso or extremities were randomized to receive BACC or AG, where the mean total BACC dosage was 234.8 cm^2^ (range: 12.0–960.0 cm^2^). The safety endpoints assessed at each visit included: 1) treatment-emergent adverse events (TEAEs), treatment-related AEs (TRAEs), and serious AEs (SAEs); 2) changes in immunologic responses (panel reactive antibodies [PRA], anti-bovine serum albumin [BSA] antibody response [STRATA2016 only]); 3) persistence of allogeneic DNA; and 4) laboratory exam and vital signs.

**Results:**

Eighty-seven patients (86.1%) experienced TEAEs, 30 patients (29.7%) experienced TRAEs, and 16 patients (15.8%) experienced SAEs. The most frequent TEAEs reported by ≥10% of patients in the pooled analysis were pruritus (n=31, 30.7%) and blister, hypertension, and hypertrophic scar (n=11, 10.9% each). The most frequent TRAEs (≥5% of patients) were pruritus (n=13, 12.9%) and blister (n=5, 5%). The most common SAEs were transplant (BACC or AG) failure, pneumonia, and deep vein thrombosis (n=2, 2% each), where only one SAE (impaired healing of moderate severity) was possibly related to BACC. One patient (1%) discontinued the trial due to a TEAE (traumatic brain injury). Two patients (2%) experienced SAEs that led to death, neither related to BACC. The number of patients with positive PRA values that were negative at baseline were 36 (38.7%) at Day 28 and 20 (22%) at Month 3. The number of patients with reactivity to HLA I class alleles found in the BACC increased from 4 (4%) at baseline to 39 (40.9%) at Day 28, then decreased to 22 (24.2%) at Month 3. No persistence of allogeneic DNA from the BACC was detected.

**Conclusions:**

BACC is well tolerated and is not associated with any unexpected SAEs or TEAEs. The safety profile at BACC treatment sites is similar to that at AG treatment sites. Thus, BACC may offer a safe alternative to autografting for the treatment of DPT burns.

